# Prediction of Microporosity in Complex Thin-Wall Castings with the Dimensionless Niyama Criterion

**DOI:** 10.3390/ma6051789

**Published:** 2013-05-07

**Authors:** Maodong Kang, Haiyan Gao, Jun Wang, Lishibao Ling, Baode Sun

**Affiliations:** The State Key Laboratory of Metal Matrix Composites, Shanghai Jiao Tong University, Shanghai 200240, China; E-Mails: kangmd518@sjtu.edu.cn (M.K.); nealllm@sjtu.edu.cn (L.L.); bdsun@sjtu.edu.cn (B.S.)

**Keywords:** nickel-based superalloy, complex thin-wall casting, microporosity, prediction

## Abstract

The dimensionless Niyama criterion was used to predict the formation of microporosity in nickel-based superalloy casting, which extended the model application from a simple plate casting to complex thin-wall superalloy casting. The physical characteristics of the superalloy were calculated by JMatPro software. The relation between the volume percentage of microporosity and the dimensionless Niyama values were constructed. Quantitative metallographic measurements of the microporosity of the practical thin-wall casting were carried out. The prediction agreed well with the experiment in general, except for some thick-wall sites in the casting.

## 1. Introduction

A nickel-based superalloy always behaves well at high temperatures and has been widely used for critical structural components in aerospace and other industries for many years because of its good mechanical property balance, malleability and weld ability. Investment casting of nickel-based superalloy is often employed for complex-shaped components, such as gas turbines, blades, and rocket engines. However, solidification defects, and especially microporosity, are still the main reason for the high rejection rate of the castings. Microporosity often appears at some special sites, such as large thin-wall and variable cross-section parts in the casting. As is well acknowledged, microporosity deteriorates the fatigue, impact toughness, and tensile properties of the casting severely. The fatigue life of the casting with microporosity is only half of that without defects, as cracks often initiate at the edges of the microporosity [1]. 

Based on the local solidification conditions, a large number of studies have focused on the formation of microporosity in the past several decades [2,3,4,5,6,7,8,9,10,11]. For instance, the volume fraction of microporosity has been predicted quantitatively for a simple plate casting based on the transmission of solutes and Sievert’s law [12]. An empirical parameter $G/\sqrt{R}$ was used in Niyama criterion to reflect the relationship between pressure drop and solidification conditions, and a threshold parameter was used for microporosity prediction [13]. The criterion made good predictions for steels; however, serious errors were encountered in Al–Si alloys [14]. Furthermore, the accurate Niyama value can hardly be obtained in most situations because it often varied with alloy composition and solidification conditions. For nickel-based superalloy, J. Lecomte-Beckers introduced a microporosity prediction model [15], where the susceptibility of microporosity formation was expressed with an index, $\Delta P^{*}$, which is related to the solidification parameters and alloy properties and can be expressed by Equation (1).

(1) $$\Delta P^{*}=\frac{24\mu \beta n\tau^{3}}{\rho_{L}g}(\frac{\Delta T_{f}}{G})(\frac{dfs}{dt})$$

Where, μ is the viscosity of the liquid, β the solidification shrinkage, *n* the number of interdendritic channels, τ the tortuosity coefficient, $\rho_{L}$ the density of liquid, g the gravitational constant, $\Delta T_{f}$ the alloy solidification range, G the mushy zone thermal gradient and dfs/dt the average solidification rate. However, some shortcomings were found in its practical application. For instance, the number of interdendritic channels and tortuosity coefficient are hard to get quantitatively. Lesoult summarized the physical principles of the formation of microporosity and pointed out that the local composition of liquid, local dendritic microstructure and local pressure drop within the mushy zone were the main causes for microporosity formation [16]. Campbell found that oxide bifilms formed during turbulent pouring have a great influence on porosity. He pointed out that the interface between the non-wetted surfaces provides a location where porosity can form. Furthermore, clean metal and counter gravity pouring can reduce porosity [17].

Based on Darcy’ law, Carlson and Beckermann proposed dimensionless Niyama criterion, where local thermal conditions, melt properties, and solidification characteristics were taken into consideration; this criterion can predict feeding-related microporosity caused by shallow temperature gradients other than gas porosity [18]. Compared with the conventional Niyama criterion, the dimensionless one is more maneuverable and quantitative in predicting microporosity formation.

The objective of the present work was to extend the application of the dimensionless Niyama criterion from a simple plate casting to a complex thin-wall superalloy casting, before the ability and accuracy of the criterion were compared to the quantitative metallographic measurements.

## 2. Mathematical Model [18]

**Figure 1:**
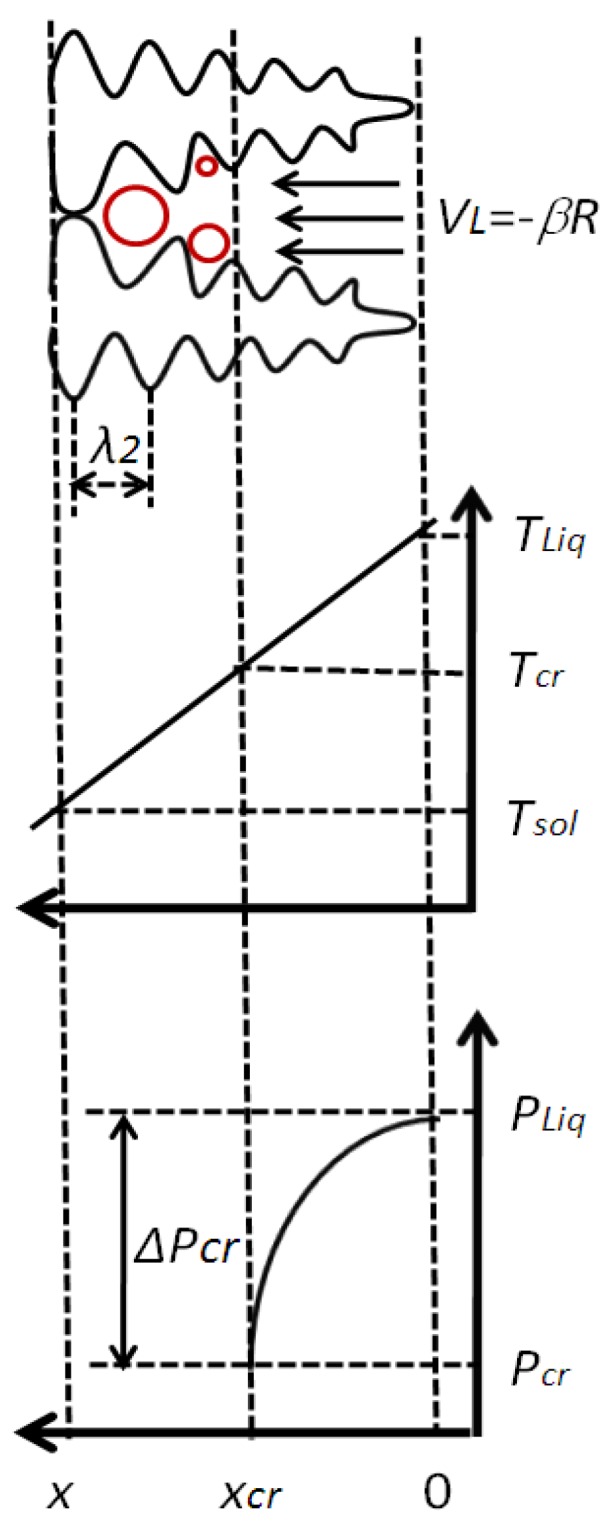
The schematic of mushy zone solidifying. Reproduced from [18].

In this section, the main evolution of the dimensionless Niyama criterion was described. The criterion was built based on the directional solidification. The schematic of the physical model is shown in Figure 1. It assumed that the liquid and solid densities ( $\rho_{L}$, $\rho_{S}$) and the cooling rate (R) are constant during solidification. The nucleation difficulty of microporosity is negligible in our research; at the moment of microporosity formation, assuming local feeding stopped and the remaining shrinkage would develop with microporosity growing.

In Figure 1, Darcy’s law can be expressed as: *f_L_V_L_* = −*KdP*/(*μdx*), where, $f_{L}$ is the liquid volume fraction, $V_{L}$ the liquid velocity, P the melt pressure, and *x* the spatial coordinate. The permeability *K* in the mushy zone is determined by the Kozeny–Carman equation *K* = *λ*_2_$f_{L}^{3}$/[180(1 − *f_L_*)], where $\lambda_{2}$ is the secondary dendrite arm spacing (SDAS).

Based on previous assumptions, shrinkage velocity in the interdendritic zone can be expressed as: *V*_L_ = −*βR*/*G*, then, $dP/dx=\mu \beta Rf_{L}/(KG)$. As the solid fraction increases, the liquid pressure $P_{liq}$ of the dendrite spacing decreases; when it reaches some critical pressure $P_{cr}$, the shrinkage porosity will form. The critical pressure is determined by the pressure inside the porosity and the capillary pressure according to the Young–Laplace equation. In the vacuum-melted superalloy, dissolved gas is very limited; the equilibrium of the Young–Laplace equation of the microporosity should be determined by the capillary pressure. Therefore, $P_{cr}$ can be simplified to the surface tension between the microporosity and the surrounding liquid metal: *P*_cr_ = −*P_σ_* = −2*σ*/*r*_0_, where σ is the surface tension and the $r_{0}$ is the initial radius curvature at microporosity formation. Assuming the constant viscosity, temperature gradient and cooling rate, the formation of microporosity in the mushy zone can be determined by integrating:

(2) $$\Delta P_{cr}=\int_{P_{cr}}^{P_{Liq}}dP=\int_{x_{cr}}^{0}\frac{\mu \beta Rf_{L}}{KG}dx=\int_{T_{cr}}^{T_{Liq}}\frac{\mu \beta Rf_{L}}{KG}\frac{dx}{dT}dT=\frac{\mu \beta R}{G^{2}}\int_{f_{L,cr}}^{1}\frac{f_{L}}{K}\frac{dR}{df_{L}}df_{L}$$

where $x_{cr}$, $T_{cr}$ and $f_{L,cr}$ are the critical position, critical temperature and critical liquid fraction, respectively. Equation (2) can be rewritten by dimensionless temperature parameter $\theta =(T-T_{Sol})/\Delta T_{f}$. $T_{Liq}$, $T_{Sol}$ are the liquid temperature and solid temperature, respectively. So,

(3) $$\Delta P_{cr}=\frac{\mu \beta \Delta T_{f}}{\lambda_{2}^{2}}\frac{R}{G^{2}}I(f_{L,cr})$$

Where

(4) $$I(f_{L,cr})=\int_{f_{L,cr}}^{1}180\frac{{\left(1-f_{L}\right)}^{2}}{f_{L}^{2}}\frac{d\theta }{df_{L}}df_{L}$$

By the solid fraction-temperature curve, the integral can be evaluated and the dimensionless Niyama criterion $Ny^{*}$ can be presented as:

(5) $$Ny^{*}=\sqrt{I(f_{L,cr})}=\frac{G\lambda_{2}\sqrt{\Delta P_{cr}}}{\sqrt{\mu \beta \Delta T_{f}R}}$$

Equation (5) accounts not only for the local solidification conditions, but also for the physical characteristics of the alloy.

When shrinkage porosity forms, the critical liquid fraction can be evaluated using Equations (3) and (5). Finally, the microporosity percentage of the alloy is obtained by Equation (6).

(6) $$f_{p}=\beta f_{L,cr}$$

The advantage of the model is that the microporosity percentage can be calculated when the local solidification condition and material properties are given. According to Equations (3)–(6), the volume percentage of microporosity (*f*_p_) can be expressed as the function of threshold dimensionless Niyama values ($Ny^{*}$). For the specific casting, the $Ny^{*}$ can also be calculated directly using local solidification parameters and alloy properties, as shown in Equation (7)

(7) $$Ny^{*}=\frac{G\lambda_{2}\sqrt{\Delta P_{cr}}}{\sqrt{\mu \;\beta \Delta T\;R}}$$

Once the specific $Ny^{*}$ is obtained in the casting, the volume percentage of microporosity can be ensured by the $Ny^{*}$ − $f_{p}$ function.

## 3. Simulation

**Figure 2:**
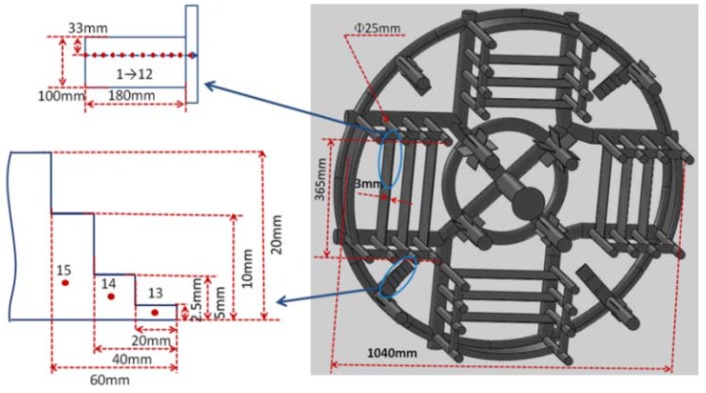
The casting geometry and the sampling positions (Numbers: 1–15).

The morphology and thickness of the complex thin-wall casting are shown in Figure 2. Two widely used softwares, JMatPro and ProCAST, were employed to calculate the parameters in the prediction. The curves of melt density-temperature and solid fraction-temperature were built by JMatPro. The criterion function ($Ny^{*}$ − $f_{p}$) of the nickel-based superalloy were obtained using Equations (3)–(6). In order to calculate the specific threshold Niyama value (Equation 7) for different sites in the nickel-based superalloy casting, thermophyscial parameters, such as liquid dynamic viscosity, freezing range, and density were also calculated using the JMatPro package.

Commercial ProCAST software was used for the solidification simulation of the casting. Under normal circumstances, fine mesh has high precision in calculation. However, excessively fine mesh consumes a great deal of time and computer resources. In this simulation, different grinding sizes were taken to optimize the calculation, 5 mm grid for pouring system and 1 mm for the thin-wall casting. The thinnest parts of the complex thin-wall casting are about 3 mm, so it is reasonable to simulate the solidification process of the thin section with 1 mm mesh size. In ProCAST calculations, the shell was considered made from refractory-fused silica with a thickness of 15 mm. It is assumed that: (1) the shell was filled with the melt at 1500 °C at the speed of 20 kg/s; (2) the initial temperature of the shell kept constant at 1000 °C before pouring; (3) the heat transfer coefficient between melt and shell was constant at 1000 W/m^2^·K [19]; and (4) the radiation from shell to surrounding environment was a constant emissivity of 0.4 without considering heat convection [20]. The calculation consumed about one day at HP workstations with 4 CPU cores and 4G memories. When ProCAST calculation finished, the temperature gradient and the cooling curve of different sites in the casting were collected directly by visual cast module of the software. The cooling rate at each site in the casting was gotten by the cooling curve.

**Figure 3:**
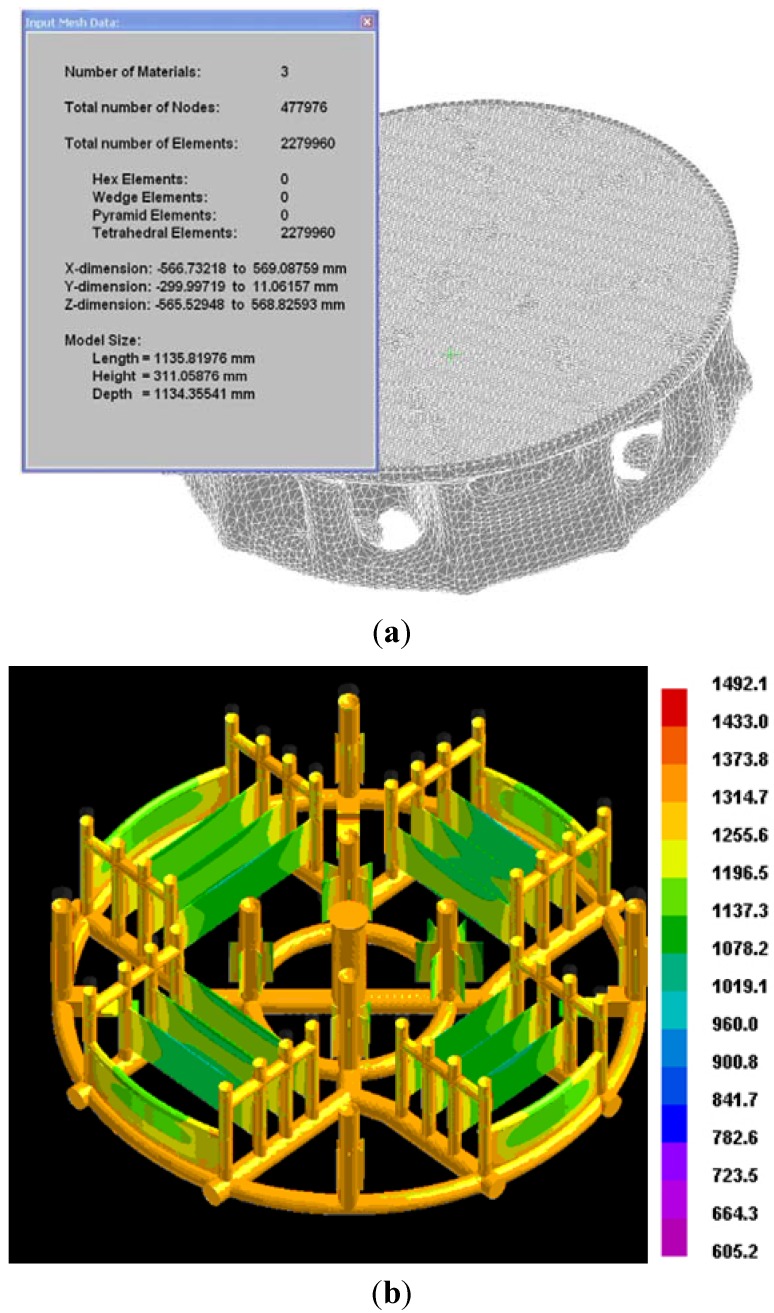
Finite element mesh and the temperature profile of the casting. (**a**) Digitized casting with shell; (**b**) Temperature field.

The corresponding mesh was generated by meshCAST (a mode of ProCAST) (see Figure 3a). The temperature profile (Figure 3b) in the stepwise parts and thin-wall parts is approximately directional, so it is suitable for the use of dimensionless criterion.

The SDAS was determined using the research of Fisher and Kurz [21], the function between SDAS and the cooling rate is shown in Equation (8).

(8) $$\lambda_{2}=C_{\lambda }R^{-1/3}$$

According to previous experimental results, the SDAS of the specimens were measured quantitatively and the relation between SDAS and the cooling rate was established by linear fitting. Then, the coefficient ($C_{\lambda }$) can be confirmed.

The dimensionless threshold Niyama values in Equation (7) were calculated and then used to predict the volume percentage of microporosity by the function of $Ny^{*}$ − $f_{p}$ (the dimensionless Niyama criterion).

## 4. Experimental

To examine the validity of the dimensionless Niyama criterion, the commercial nickel-based superalloy was used for the experiment. The chemical composition of the alloy was tested by Inductively Coupled Plasma (Model: ICAP 6000 Radial) and high frequency infrared ray carbon sulfur analyzer (Model: CS-206), and the result was shown in Table 1. Ceramic shells were made from multi-component slurries, including a fine mesh refractory filler system and a colloidal binder system. 

**Table 1:** Chemical composition of nickel-based superalloy.

Elements	C	Cr	Ni	Co	Mo	Al	Ti	Fe	Nb	Ta
Composition (wt %)	0.06	19.43	52.09	0.18	3.15	0.41	1.06	19.08	4.36	0.08

**Figure 4:**
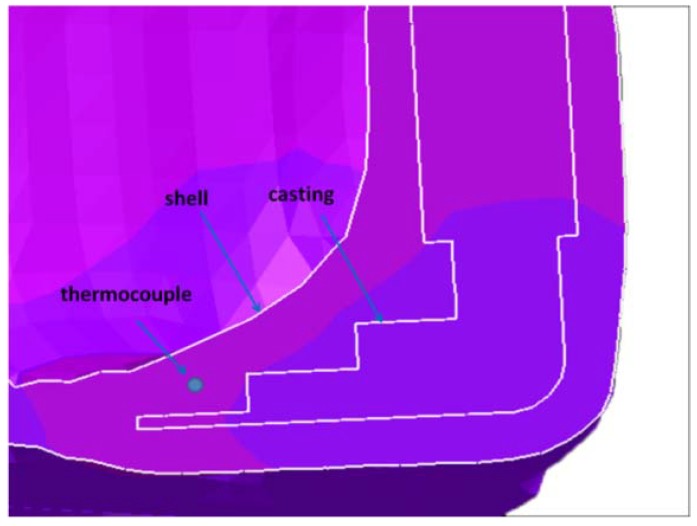
The relative position of thermocouple.

The shell was heated to 1000 °C and held for 20 h before pouring. The superalloy was melted in a vacuum investment casting system, and then poured into the preheated ceramic shell under gravity. To verify the accuracy of parameters given by ProCAST, thermocouples were placed in the ceramic shell to monitor the temperature at some typical sites, as shown in Figure 4. Other experimental conditions were common with the ProCAST simulation, including pouring temperature, filling velocity, *etc.*

The microporosity of the casting was investigated using Zeiss optical microscopy. Specimens were cut from the casting and then ground, polished and observed. Microporosity measurement was carried out using DT2000 commercial image analysis system. The percentage of microporosity was characterized within an area of 1 mm × 1 mm (uniform size with simulation) at the magnification of 50, and the maximum value was used as the measurement results.

## 5. Results and Discussion

The complex thin-wall casting is mainly composed of thin plates, stepwise parts and the well-designed pouring system. The temperature field is similar to directional solidification on the basis of ProCAST calculation (see Figure 3b), so the conditions meet the criterion’s requirements.

### 5.1. The Prediction Function for Nickel-Based Superalloy

**Figure 5:**
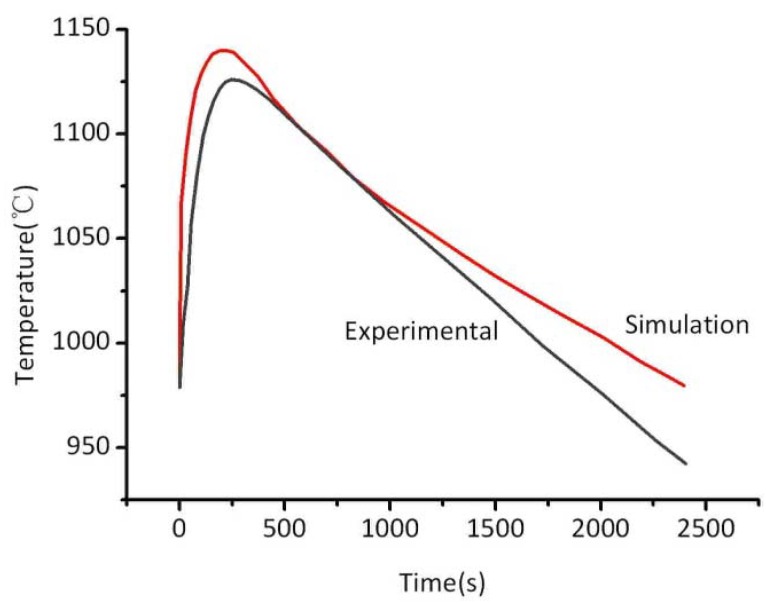
Comparison of the prediction and experimental cooling curve.

Figure 5 compares the cooling curve monitored by the thermocouple with the ProCAST simulation. The calculated profile is in good agreement with the experimental. Similarly, the simulated temperature of the internal casting can be treated as the real one in calculating the cooling rate, and this method has been used to other positions in the following calculation.

**Figure 6:**
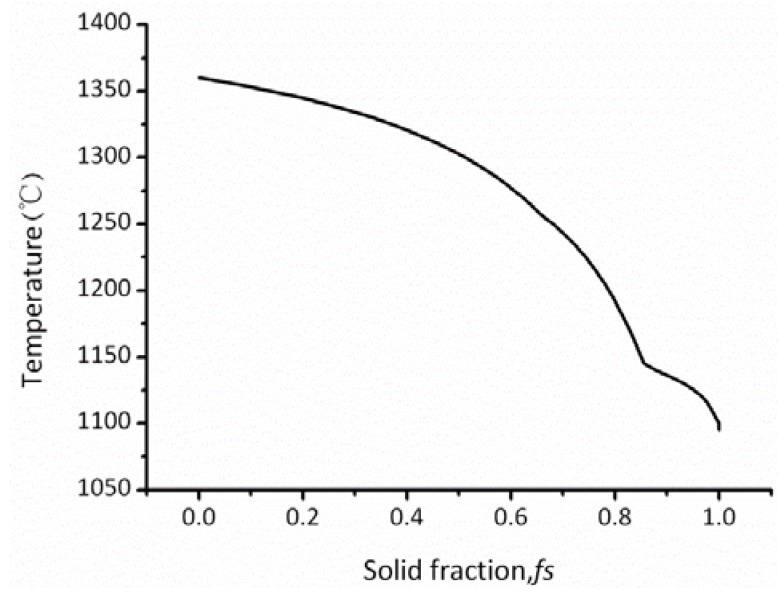
Solid fraction curve of the superalloy with temperature.

**Figure 7:**
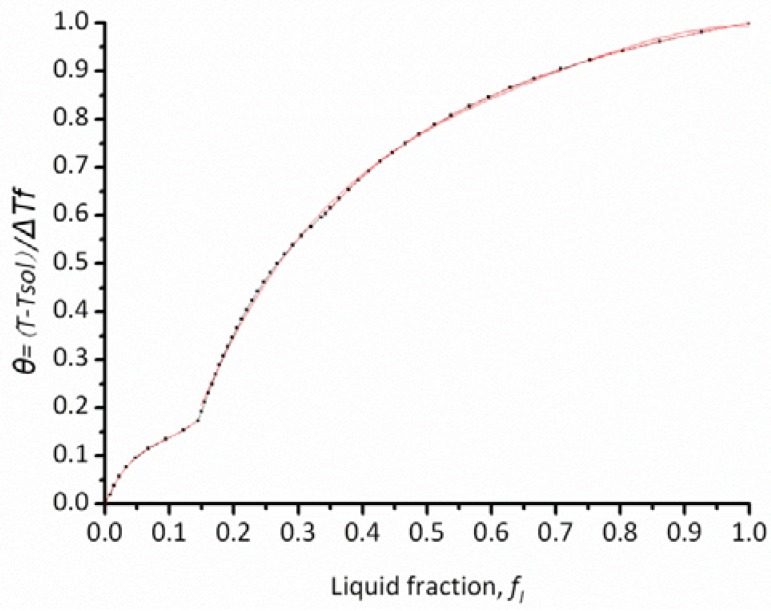
The function between liquid fraction and dimensionless temperature.

Figure 6 shows the solid fraction–temperature curve of the superalloy calculated by the JMatPro package. After changing the form of the temperature in Figure 6, the dimensionless temperature curves were obtained for nickel-based superalloy in Figure 7.

**Figure 8:**
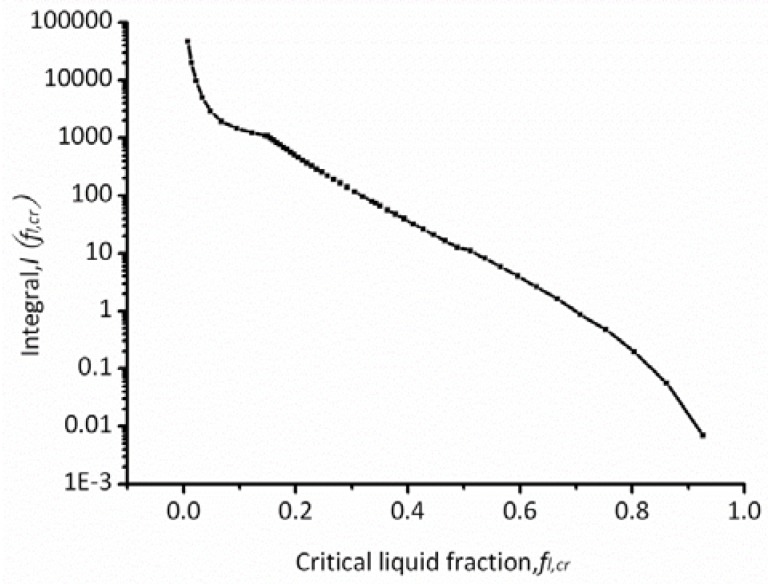
Variation in the integral with critical liquid fraction.

In order to get the slope ($m=d\theta /df_{l}$) of Figure 7, the fitting equations are given in Table 2. The fitting curves are also given in Figure 7 (red lines). The integration in Equation (4) can be evaluated numerically using commercial computing software, and Figure 8 illustrates the resulting relationship between $I(f_{l,cr})$ and the critical liquid fraction, $f_{l,cr}$, for the used nickel-based superalloy.

**Table 2:** The fitting relationship of *θ* and *f_L_*.

Liquid fraction (*f_L_*)	The relationship of *θ* and *f_L_*
0.75248–1	$\theta =0.23+1.65f_{L}-1.22{f_{L}}^{2}+0.34{f_{L}}^{3}$
0.51169–0.75248	$\theta =-0.115+3.1f_{L}-3.3{f_{L}}^{2}+1.344{f_{L}}^{3}$
0.14456–0.51169	$\theta =-0.4284+5.54f_{L}-10.04{f_{L}}^{2}+9.32{f_{L}}^{3}-3.27{f_{L}}^{4}$
0–0.14456	$\theta =3.25f_{L}-32.84{f_{L}}^{2}+172.72{f_{L}}^{3}-300.82{f_{L}}^{4}$

**Figure 9:**
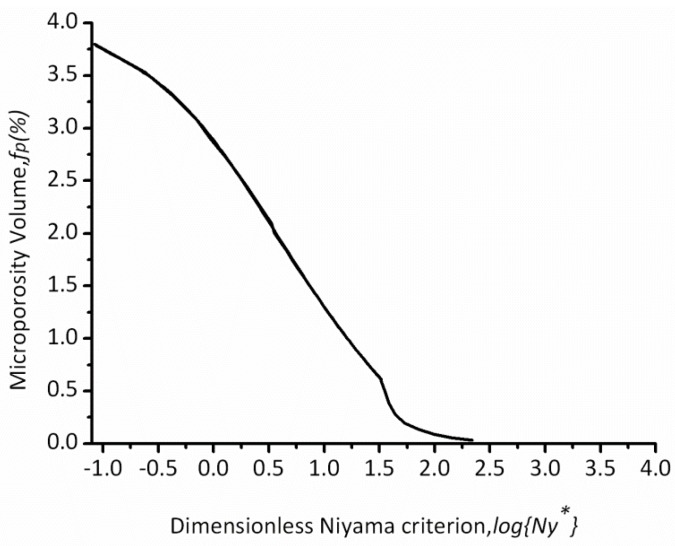
Predicted microporosity volume as a function of log [$Ny^{*}$] form.

Through a combination of Figure 8 with Equations (6) and (7), the relation between microporosity volume fraction, $f_{p}$, and the dimensionless Niyama values, $Ny^{*}$ can be obtained as shown in Figure 9. The volume fraction of microporosity decreases with the increase of the dimensionless Niyama value and close to zero when the Niyama value increases to a certain extent, which is similar to that of WCB steel [18].

### 5.2. Microporosity Prediction

Parameters in Equation (7) should be determined before predicting microporosity in the specific sites of the casting. They can be acquired one by one, as follows.

#### 5.2.1. Secondary Dendritic Arm Spacing, $\lambda_{2}$

**Figure 10:**
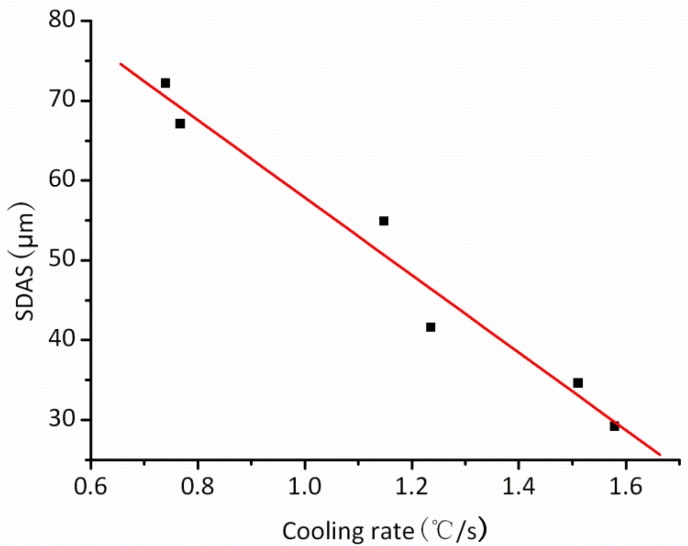
The relation between SDAS and cooling rate.

In Equation (8), the coefficient, $C_{\lambda }$, is a constant related to the alloy. Figure 10 shows the relation between SDAS and the cooling rate for the superalloy used in the experiment. The scattered data point in Figure 10 was obtained by previous experimental results, and the straight line was the linear fitting of the experimental data. Then, the value of $C_{\lambda }$ was easily obtained as 4.8 × 10^−5^ m·(°C/s)^1/3^. Thus, the SDAS of the specific sites in the casting can be calculated using Equation (8).

#### 5.2.2. Alloy solidification range, $\Delta T_{f}$ and solidification shrinkage rate, β

**Figure 11:**
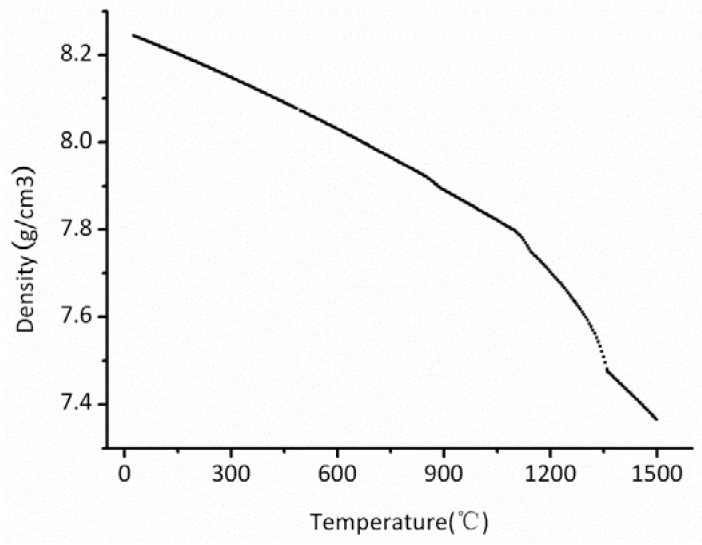
Calculated density of the nickel-based superalloy at different temperatures.

JMatPro package calculation revealed that the liquid temperature of the used superalloy is about 1360 °C, and the superalloy has a freezing range of 260 °C. The calculated density–temperature curve was given in Figure 11, and the solid/liquid densities are about 7.80 g/cm^3^ and 7.48 g/cm^3^, respectively. The solidification shrinkage rate can then be calculated about 0.04.

#### 5.2.3. Cooling rate, R and thermal gradient, G

The slope of the cooling curve of the casting was used as the cooling rate, similar to Section 5.1; and the thermal gradient was directly extracted from the visual cast module of the ProCAST package.

#### 5.2.4. Critical pressure, $\Delta P_{cr}$ and liquid dynamic viscosity, μ

The parameter $\Delta P_{cr}$ was often set at 0.1 MPa by assuming that the melt pressure was equal to the atmospheric pressure at the liquid temperature [18]. Besides, the liquid dynamic viscosity was assumed to be constant as 8.9 mPa·s in the calculation.

**Figure 12:**
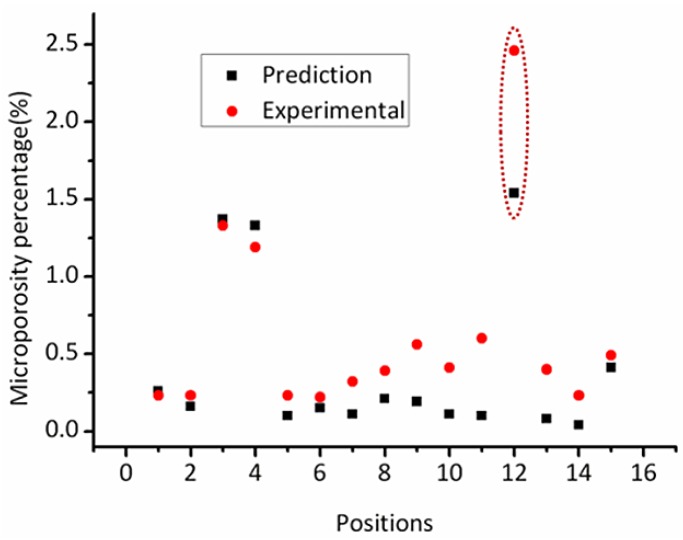
The experimental and the prediction microporosity percentage in different positions (Number: 1–15).

Two steps must be taken to predict the microporosity of the complex thin-wall casting. Firstly, the dimensionless Niyama values were calculated by Equation (7); secondly, the percentage of the microporosity volume was obtained by Figure 9. For a better comparison, the predicted and experimental results of the quantitative metallographic analysis were given in Figure 12. 

**Figure 13:**
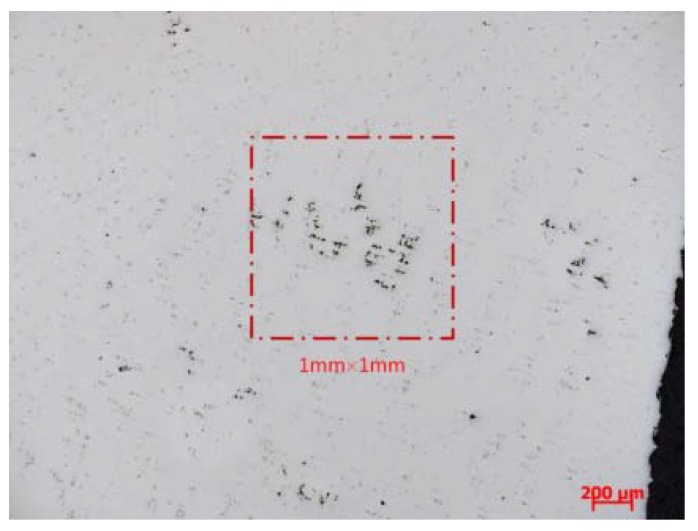
Optical view of microporosity, and the content of microporosity, 1.26%.

In general, the tendencies of the prediction results are in reasonable agreement with the experimental results. For example, Figure 13 shows a typical optical view of microporosity at the position of NUM 3, where about 1.26% interdendritic microporosity exists; and the experimental result agrees well with the dimensionless Niyama criterion prediction (1.25%).

However, the predicted is a bit lower than the measured, which may be caused by the neglect of gas evolution during solidification. According to Campbell’s research, the melt entrain bifilms because turbulent pouring in vacuum (dilute air), and unfolding of bifilms, which may occlude gases, will produce porosity; criteria functions cannot predict porosity originating from bubbles and mold gases caused by a poor pouring system [22].Though the melt had been refined, some gas emerged during pouring (melt–mold interaction) or solidification, which led to a rise in the volume of microporosity. The gas content of the experiments is shown in Table 3. The total gas content of the casting increases by 51% over the base metal.

**Table 3:** Gas content of the base metal and the casting.

Elements	O	N	H
Base metal (%)	0.0009	0.0040	0.00012
Casting (%)	0.0045	0.0030	0.00007

Moreover, the difference between predicted and measured microporosity in the position of NUM12 is vast. In the experiment, the position was selected to specify the application of the dimensionless Niyama criterion. Thickness of NUM12 is much larger than other points and the local solidification condition cannot meet the requirement of directional solidification. In fact, it formed hot spots (see Figure 3b) in the thick section and was beyond the application scope of the criterion. The results show that the dimensionless Niyama criterion is not suitable for shrinkage prediction in thick structures, because those sections often form isolated liquid pools.

## 6. Conclusions

(1) Application of the dimensionless Niyama criterion in predicting the volume percentage of microporosity in a nickel-based superalloy casting was investigated.

(2) The relation between the dimensionless Niyama values and the microporosity of the superalloy has been obtained; the prediction results show reasonable agreement but under-predict those of the experiments concerning porosity content in the complex thin-wall casting.

(3) The poor prediction results in the thick parts of the casting revealed that the criterion was not suitable for the shrinkage prediction of isolated liquid pools.
